# The impact of complications on a programme of enhanced recovery in colorectal surgery

**DOI:** 10.1186/s12893-018-0390-7

**Published:** 2018-08-16

**Authors:** Giovanni D. Tebala, Antonio Gallucci, Abdul Q. Khan

**Affiliations:** 1 Colorectal Team, Noble’s Hospital, Douglas, Isle of Man UK; 2 East Kent Hospitals University NHS Foundation Trust, William Harvey Hospital, Kennington Rd, Willesborough, Ashford, Kent, TN24 0LZ UK

**Keywords:** Colorectal surgery, Enhanced recovery, Laparoscopic colorectal surgery, Complications

## Abstract

**Background:**

The advantages of Enhanced Recovery (ER) programmes are well known, in terms of improved overall experience of the patients, which associates with low morbidity and reduced length of stay. As a result, the pattern of morbidity is changing and some patients may develop complications after discharge. Aim of this work was to evaluate the impact of morbidity and related outcomes such as unplanned readmission and reoperation rate on an ER programme in colorectal surgery.

**Methods:**

Prospectively collected clinical data of patients who underwent colorectal resection have been retrospectively analysed. Endpoints were: 90-day mortality and morbidity, length of hospital stay (LOS) and rate of unplanned readmissions and reoperations.

**Results:**

Mortality and morbidity did not change in the analysed period, but LOS reduced significantly. Main determinant of postoperative LOS was the type of surgical approach, laparoscopy being associated with earlier discharge. LOS was longer in patients who developed complications. Morbidity and reoperation rate were significantly higher in patients discharged after day 4. Majority of complications happened in patients who were still in the hospital. However, the few patients who developed complications after discharge did not have a worse outcome if compared to those who had complications in hospital.

**Conclusions:**

ER protocols must become integral part of the perioperative management of colorectal patients. ER and laparoscopy have a synergic effect to improve the postoperative recovery and reduce morbidity. Early discharge of patients does not affect the outcome of postoperative complications.

## Background

The concept of surgical Fast Track has been proposed in the 90s by Henrik Kehlet [1, 2] as a consequence of the increased demand for better efficacy and reduced invasiveness of surgery by patients and surgeons. It entails applying the most recent scientific evidence to the perioperative management of surgical patients, in order to improve their postoperative recovery and, as a consequence, reduce their hospital stay [3–5]. Unfortunately, at least at the beginning, this last factor has been considered by many as the real aim of the new protocols. Also the name initially chosen, “Fast Track”, recalled mostly an early (but probably unsafe) discharge of a surgical patient, without highlighting the real benefits. For this reason many surgeons were – and in many cases still are – concerned of the possible medico-legal consequences in case of complications and many patients considered it only a way to reduce the costs of healthcare. We believe that the recent definition of “Enhanced Recovery” (ER) is more consistent with the spirit of the new perioperative protocols as it evokes clear advantages for the patients and the surgeon. ER protocols aim at optimising the postoperative recovery as they “reduce surgical stress, maintain postoperative physiological function, and enhance mobilisation after surgery” [6].

ER concepts found a wide application in colorectal surgery and specific guidelines have already been published [6–8]. However, substantial differences still exist on how the guidelines are applied locally, according to the preferences of the surgeon and the constant evolution of evidence [9, 10].

As a consequence of the improvement yielded by ER programmes, the pattern of morbidity is changing and, although general morbidity is decreasing, more patients may develop complications after discharge. Is this going to affect their outcome?

In this work we will discuss of the impact of complications on an enhanced recovery in colorectal surgery programme and we will evaluate our specific experience, matured in more than 4 years at the Colorectal Team of the Noble’s Hospital, Isle of Man.

## Methods

Prospectively collected and anonymised demographic and clinical data of patients who underwent colorectal resections under an ER programme from March 2013 to April 2017 at the Colorectal Unit of the Noble’s Hospital (Isle of Man) have been analysed with a statistical package (IBM SPSS for Mac). Noble’s Hospital is a 314-bed teaching hospital serving a population of about 90,000. Our ER protocol, which we implemented in 2013, has already been described [5]. Briefly, after discussion of his or her case at the colorectal multidisciplinary team meeting, the patient meets the consultant surgeon and the colorectal specialist nurse in the clinic. In this first meeting the patient is informed of his/her diagnosis and the treatment plan is offered, including recruitment into the ER programme for those who are considered for surgery. Subsequently, the patient meets the consultant anaesthetist and the preoperative assessment nurse, and the ER protocol is further explained and discussed in all its components. Specific perioperative issues, including comorbidities, nutritional requirements, anaesthesia and postoperative analgesia, are discussed and addressed at this juncture, and the discharge is accurately planned well before the surgery. The patient is suggested to have specific carbohydrate rich drinks (Preload, Vitaflo Int Ltd., Liverpool, UK) on the day before the operation and at 6 am on the day of surgery and to fast to solid food for 6 h before the operation and to liquids for 2 h. We adopt a simple selective bowel preparation protocol. Patients scheduled for proximal colectomies (right or extended right colectomy) do not have any bowel preparation. Those listed for distal colectomies (left or sigmoid colectomy, anterior resection, abdominoperineal resection - APR) have a standard preparation with macrogol (Moviprep, Norgine, Harefield, UK). Patients with proximal colostomy scheduled for distal colectomy have only a phosphate enema on the morning of the operation. All patients have deep vein thrombosis prophylaxis with enoxaparin and thrombo-embolic deterrent stockings. No premedication is routinely administered. Intraoperative anaesthesiological management is based on: (a) fluid volume based on cardiac output, generally checked with non-invasive monitoring, (b) prevention of hypothermia with warm fluid and warm air blankets, (c) short-acting anaesthetic agents. Use of opiates is minimal, in particular in the postoperative period. Postoperative analgesia is achieved with paracetamol in laparoscopic colectomies and with epidural analgesia with bupivacaine and fentanyl in patients who had a laparotomic operation. Antibiotic prophylaxis is routinely achieved with cefuroxime and metronidazole 1 h before the operation and three more doses in the postoperative period. Laparoscopy is offered to every patient, irrespective of their age, comorbidities, BMI, kind and location of surgery, if a specifically skilled and qualified consultant is available and no specific contraindication to laparoscopy exists. We routinely fashion a covering loop ileostomy in all patients undergoing rectal resections below the peritoneal pelvic reflection, to be reversed as soon as possible and anyway within 3 months of surgery, after visual (flexible sigmoidoscopy) and radiological (water-soluble enema) check of the anastomosis. No nasogastric tube is routinely used, unless the patient needs gastric decompression during surgery; in this case the tube is removed at the end of the operation. The bladder catheter is removed as soon as the patient is able to walk to the toilet unaided, and this usually happens within 24 h after the operation. Use of drains is selective but our policy on their use has changed quite significantly in the last years. At the beginning of this experience with the ER programme we were used to leave one drain in patients with resection above the peritoneal reflection and two drains in patients undergoing resection below the pelvic reflection. Now we do not routinely drain colonic resections (above the peritoneal reflection), but still use two pelvic drains in low anterior resections. In APRs/ELAPEs we usually leave a single transperineal drain in the pelvic cavity and suture close the pelvic peritoneum. Drains are ideally removed in day 1 or 2. Patients are allowed to drink immediately after surgery and to solid food as soon as they are comfortable and tolerate oral fluids (ideally a couple of hours after surgery). They are encouraged and helped to mobilise as soon as possible, with the help of the physiotherapist if needed. The patient is discharged when the following criteria are met: (a) he/she is passing flatus or the stoma is working, (b) he/she is tolerating food, (c) the pain is well controlled with oral analgesia, (d) there are no signs of ongoing complication, (e) all the social arrangements are in place to allow a safe return home. If the patient’s clinical signs (blood pressure, heart rate, respiratory rate, temperature, pain, neurologic status) are fine we do not check his/her blood routinely. After discharge, the patient is followed up telephonically on daily basis by the specialist nurse and is checked in the clinic within 2 weeks, to make sure he/she is recovering well and to discuss the results of histology.

Endpoints of this study are: 90-day mortality and morbidity (Clavien > 2), length of hospital stay (LOS), number of unplanned readmissions within 90 days, number of unplanned reoperations within 90 days.

Data will be presented as mean ± standard deviation, median and range or number of cases and percentage. Comparison of categorical variables and frequencies between groups was performed with the Pearson’s Chi-square test. Comparison between groups of the only numerical variable (LOS) was performed with the Mann-Whitney’s U-test due to non-normal distribution. Independent variables have been identified with logistic or linear regression analysis performed on the whole series and on the subgroup of elective patients. *P* values less than 0.05 were considered to be significant. However, when the difference between two groups did not reach statistical significance due to the size of the sample but showed an interesting and clinically significant trend, this has been highlighted.

All the patients involved in this study gave full informed consent to the operation and to be recruited in our ER programme. The implementation of the ER programme in colorectal surgery has been approved by the Medical Director of the Noble’s Hospital. The Local Research Ethical Committee of the Noble’s Hospital confirmed that formal approval was not necessary due to the retrospective nature of the study. Formal written consent to participate in the study was not obtained from participants because the study reports the results of a retrospective analysis of anonymised clinical data.

## Results

In the analysed period, 198 patients (92 women and 106 men, aged 68.7 ± 11.4) underwent colorectal resections within an ER programme. Demographic and clinical data and results of univariate analysis are reported in Tables 1 and 2. Tables 3 and 4 report the results of multivariate analysis on the whole series and on the subgroups of elective patients.

**Table 1:** Demographics and clinical data and comparison between groups (univariate analysis) (*p*-values highlighted in bold are to be considered statistically and/or clinically significant)

	Total	Mortality	Morbidity	Leak	Medical complications	Postop LOS mean ± SD (median)	Patients discharged within day4	Readmission	Reoperation
Total	198	6 (3.0%)	28 (14.1%)	9 (4.5%)	7 (3.5%)	9.5 ± 12.1 (6)	68 (34.3%)	14 (7.1%)	18 (9.1%)
F M	92 (46.5%) 106 (53.5%)	5 (5.4%) 1 (0.9%) *p* = 0.066	18 (19.6%) 10 (9.4%) *p* = 0.041	3 (3.3%) 6 (5.7%) *p* = 0.419	5 (5.4%) 2 (1.9%) *p* = 0.178	8.7 ± 10.3 (6) 10.2 ± 13.4 (6) *p* = 0.713	31 (33.7%) 37 (34.9%) *p* = 0.858	8 (8.7%) 6 (5.7%) *p* = 0.406	11 (12.0%) 7 (6.6%) *p* = 0.191
<65yo >65yo	92 (46.5%) 106 (53.5%)	2 (2.2%) 4 (3.8%) *p* = 0.513	15 (16.3%) 13 (12.3%) *p* = 0.416	2 (2.2%) 7 (6.6%) *p* = 0.136	4 (4.3%) 3 (2.8%) *p* = 0.564	9.1 ± 14.5 (6) 9.8 ± 9.5 (7) *p* = 0.082	34 (37.0%) 34 (32.1%) *p* = 0.471	5 (5.4%) 9 (8.5%) *p* = 0.403	8 (8.7%) 10 (9.4%) *p* = 0.857
Elective Emergency	166 (83.8%) 32 (16.2%)	4 (2.4%) 2 (6.3%) *p* = 0.246	23 (13.9%) 5 (15.6%) *p* = 0.793	8 (4.8%) 1 (3.1%) *p* = 0.674	5 (3.0%) 2 (6.3%) *p* = 0.364	7.8 ± 7.6 (6) 18.3 ± 22.9 (13) ***p*** ** = 0.000**	63 (38.0%) 5 (15.6%) ***p*** ** = 0.015**	13 (7.8%) 1 (3.1%) *p* = 0.342	17 (10.2%) 1 (3.1%) *p* = 0.200
Cancer Other	146 (73.7%) 52 (26.3%)	5 (3.4%) 1 (1.9%) *p* = 0.588	20 (13.7%) 8 (15.4%) *p* = 0.764	8 (5.5%) 1 (1.9%) *p* = 0.290	5 (3.4%) 2 (3.8%) *p* = 0.888	8.4 ± 9.9 (6) 12.7 ± 16.4 (9) ***p*** ** = 0.001**	58 (39.7%) 10 (19.2%) ***p*** ** = 0.008**	10 (6.8%) 4 (7.7%) *p* = 0.839	14 (9.6%) 4 (7.7%) *p* = 0.683
Laparosc Laparot	124 (62.6%) 74 (37.4%)	2 (1.6%) 4 (5.4%) *p* = 0.132	14 (11.3%) 14 (18.9%) *p* = 0.136	3 (2.4%) 6 (8.1%) ***p*** **= 0.063**	4 (3.2%) 3 (4.1%) *p* = 0.760	6.4 ± 5.8 (5) 14.7 ± 17.1 (10) ***p*** ** = 0.000**	61 (49.2%) 7 (9.5%) ***p*** ** = 0.000**	8 (6.5%) 6 (8.1%) *p* = 0.660	12 (9.7%) 6 (8.1%) *p* = 0.710
Prox resect Distal resec Rectal res Total colec Other	57 (28.8%) 58 (29.3%) 63 (31.8%) 9 (4.5%) 11 (5.6%)	0 3 (5.2%) 2 (3.2%) 0 1 (9.1%) *p* = 0.361	3 (5.3%) 10 (17.2%) 13 (20.6%) 1 (11.1%) 1 (9.1%) *p* = 0.156	0 3 (5.2%) 6 (9.5%) 0 0 *p* = 0.120	2 (3.5%) 4 (6.9%) 1 (1.6%) 0 0 *p* = 0.500	7.9 ± 9.2 (5) 10.0 ± 12.0 (7) 9.0 ± 7.9 (7) 10.3 ± 5.5 (10) 17.0 ± 32.9 (7) *p* = 0.189	24 (42.1%) 18 (31.0%) 21 (33.3%) 1 (11.1%) 4 (36.4%) *p* = 0.405	1 (1.8%) 3 (5.2%) 8 (12.7%) 1 (11.1%) 1 (9.1%) *p* = 0.192	1 (1.8%) 5 (8.6%) 11 (17.5%) 1 (11.1%) 0 ***p*** ** = 0.037**
2013 (10 m) 2014 2015 2016 2017 (4 m)	27 (13.6%) 57 (28.8%) 48 (24.2%) 47 (23.7%) 19 (9.6%)	1 (3.7%) 1 (1.8%) 2 (4.2%) 1 (2.1%) 1 (5.3%) *p* = 0.907	4 (14.3%) 7 (12.3%) 7 (14.6%) 6 (12.8%) 4 (21.1%) *p* = 0.910	1 (3.7%) 1 (1.8%) 3 (6.3%) 2 (4.3%) 2 (10.5%) *p* = 0.564	2 (7.4%) 2 (3.5%) 1 (2.1%) 2 (4.3%) 0 *p* = 0.690	13.5 ± 16.0 (7) 10.0 ± 10.7 (7) 9.2 ± 16.3 (6) 7.5 ± 6.4 (6) 8.1 ± 6.1 (6) *p* = 0.126	4 (14.8%) 14 (24.6%) 20 (41.7%) 22 (46.8%) 8 (42.1%) ***p*** ** = 0.018**	3 (11.1%) 1 (1.8%) 8 (16.7%) 2 (4.3%) 0 ***p*** ** = 0.018**	3 (11.1%) 5 (8.8%) 3 (6.3%) 3 (6.4%) 4 (21.1%) *p* = 0.365
LOS > 4 LOS ≤ 4	130 (65.7%) 68 (34.3%)		26 (20.0%) 2 (2.9%) ***p*** **= 0.001**	8 (6.2%) 1 (1.5%) *p* = 0.133	7 (5.4%) 0 ***p*** **= 0.051**	12.9 ± 13.8 (8) 3.1 ± 0.8 (3)		11 (8.5%) 3 (4.4%) *p* = 0.291	17 (13.1%) 1 (1.5%) ***p*** ** = 0.007**
Complic Non-compl	28 (14.1%) 160 (85.9%)					17.8 ± 16.0 (13.5) 8.1 ± 10.8 (6) ***p*** ** = 0.000**	2 (7.1%) 66 (38.8%) ***p*** ** = 0.001**

**Table 2:** Operations

Operation	N. (%)	Laparosc (%)	Associated colectomies
Right colectomy	42 (21.2%)	34 (81.0%)	2 sigmoid resections
Extended right colectomy	15 (7.6%)	10 (66.7%)	
Left colectomy	14 (7.1%)	12 (85.7%)	
Extended left colectomy	2 (1.0%)	0	
Sigmoid resection	25 (12.6%)	20 (80.0%)	
Hartmann	17 (8.6%)	0	
Anterior resection	51 (25.8%)	35 (68.6%)	
APR	8 (4.0%)	7 (87.5%)	
Intersphincteric proctectomy	3 (1.5%)	0	
Segmental colectomy	12 (6.0%)	0	
Total/subtotal colectomt	9 (4.5%)	6 (66.7%)	
TOTAL	198	124 (62.6%)

**Table 3:** Multivariate analysis. (a) logistic regression (b) linear regression

a.			
Endpoint	Independent variables	Odds Ratio	p
Mortality	Male gender	0.061	0.037
Morbidity	Male gender	0.344	0.019
Leak	Laparoscopic resection	0.127	0.014
Medical complications	NS		
Discharge within day4	Laparoscopic resection	18.601	0.000
Reoperations	Site of resection	2.060	0.013
Readmissions	NS		
b.			
Endpoint	Independent variables	Standardised coefficient	p
Postoperative LOS	Laparoscopic resection	−0.213	0.012
	Elective resection	−0.240	0.006
	Year	−0.161	0.016

**Table 4:** Multivariate analysis in elective cases. (a) logistic regression (b) linear regression

a.			
Endpoint	Independent variables	Odds Ratio	p
Mortality	Laparoscopic resection	0.056	0.043
Morbidity	NS		
Leak	Laparoscopic resection	0.109	0.016
Medical complications	NS		
Discharge within day4	Laparoscopic resection	21.671	0.000
	Year	1.852	0.000
Reoperations	NS		
Readmissions	NS		
b.			
Endpoint	Independent variables	Standardised coefficient	p
Postoperative LOS	Laparoscopic resection	−0.320	0.000
	Age > 65	0.240	0.002

Almost 63% of resections (and 74.7% of elective resections) were performed by laparoscopy. Conversion rate was 10.8%.

Total mortality (3%) and morbidity (14.1%) were significantly higher in women than in men. Mortality was also higher – but not significantly – in open resections and in cancer operations. Mortality and morbidity did not change significantly in the 4 years period. Medical complications happened in 3.5% of patients. General leak rate was 4.5%, higher in open than in laparoscopic resections (8.1% vs 2.4%). Risk of leak for laparoscopic operations is reduced by 90% with respect to open operations. Leak rate was also higher in distal resections and in elderly patients. Very most of patients who had a leak (8/9) have been reoperated (3 stomas + drainage, 5 re-resections).

Obviously, complications affected LOS as none of the patients who had a complication could be discharged within day 4.

LOS was shorter in elective vs emergency resections, in cancer with respect to non-cancer patients and mostly in laparoscopic vs open resections. These data were confirmed also at multivariate analysis, which demonstrated that an elective patient operated on by laparoscopy has a possibility 21 times higher than those operated on by open surgery to be discharged within day 4. Elective and laparoscopic surgery were also confirmed as independent causative variables at multivariate analysis. In particular, the laparoscopic technique has been demonstrated to be also an independent protective factor towards mortality and anastomotic morbidity.

**Fig. 1:**
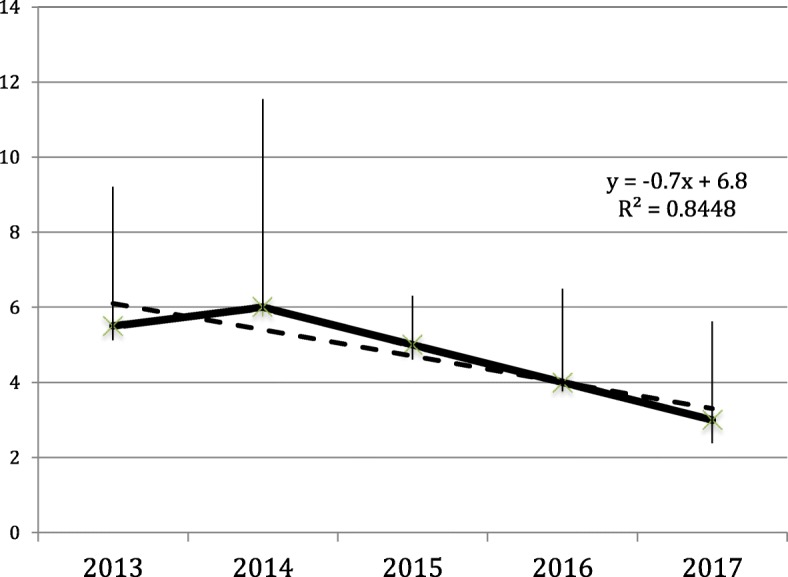
Trend of the median postoperative lenght of stay (with confidence interval of the mean to confirm the non-normal distribution) in elective non-complicated patients. The linear regression, shown by the regression line, confirms the trend to reduction of the LOS with increased experience with ER. The regression equation is reported together with the value of R^2^

Although the overall LOS did not change significantly in the analysed period, in elective non-complicated patients LOS progressively reduced from 2013 to 2017 (Fig. 1). The rate of patients discharged within day 4 increased accordingly from 2013 to 2017.

Readmission rate was not related to any of the parameters analysed. On the contrary, reoperation rate was higher in elective patients and in distal resections.

Early discharged patients had a reduced risk of readmission and reoperation with respect to those discharged after day 4.

Most complications happened in hospital, but 5 out of 28 (17.9%) patients had to be readmitted due to a complication arisen after discharge. They were all surgical complications (4 leaks, 1 rectovaginal fistula). Four out of 9 (44.4%) anastomotic leaks happened after discharge. Reoperation and mortality rates for readmitted patients were similar to those of non-readmitted ones (80% vs 61%, *p* = 0.400; 20% vs 22%, *p* = 0.932).

## Discussion

It has been demonstrated that the application of an ER protocol allows a reduction of the overall – mainly medical - morbidity [4, 11–16] and costs [4], whereas surgical morbidity is not affected.

As a consequence of the prompt and improved recovery, LOS is reduced as well [16].

It is interesting to note that the ER protocol allows a significant improvement of the results within single units, with respect to the pre-ER period [4, 14, 15, 17]. Our experience demonstrated a significant progressive reduction of LOS with increasing confidence with the ER protocol from 2013 to 2017. This trend allows us to preconize that within few years most of our patients could be possibly discharged within 24 h [18]. The implementation of the ER programme with reduced length of stay did not increase morbidity and readmission rate. On the contrary, readmission rate significantly reduced with time. Our data compare favourably with those of the UK National Bowel Cancer Audit [19].

The fear that an early discharge could increase the risk of complication has not been supported by any evidence. On the contrary, early discharge is almost invariably associated with lower morbidity and reduced risk of readmission [20]. This could suggest that an early discharge is the cause for a lower morbidity. Even if it is theoretically possible that patients recovering at home have a lower risk of hospital acquired infection, deep vein thrombosis and pulmonary embolism, the most likely explanation for the positive relation between early discharge and lower morbidity is that patients who recover well and quickly after an operation have a lower risk of developing complications and can be discharged earlier.

Our results, in accordance with those of Faiz et al. [21], demonstrated that ER is able to reduce LOS but it does not increase the risk of unplanned readmission. In our experience, the growing adherence to the ER principles permitted a progressive reduction of the readmission rate.

In the Literature, the unplanned readmission rate for ER patients is similar to non-ER patients [16, 20, 22]. There is also good evidence that the lack of adherence to ER protocol may be one of the determinants of the risk of unplanned readmission [20]. An early discharge does not increase the risk of readmission, but, on the contrary in our experience readmission rate and reoperation rate were higher in patients discharged later, as reported by other Authors [23]. These patients were probably those who could not be discharged earlier due to sluggish recovery and higher risk of complications. The fact that most of unplanned reoperations are necessary in patients discharged late – and not in patients discharged and then readmitted – allows us to infer that complications, when they happen, they give specific signs in the immediate postoperative period that can raise the clinical suspicion and contraindicate discharge. In our series, readmission rate was higher in patients with longer stay, but reoperation rate was independent by length of stay.

ER allows early identification of complications. In fact, a clinical recovery which is slower than expected and a less than prompt re-establishment of normal physiological signs (bowel sounds, full mobility…) in a patient recruited within an ER programme should raise the suspicion of an ongoing complication and prompt the request for investigations. As a matter of fact, in our series, despite a low median LOS more than 80% of complications happened during the main admission, and only 18% of complications became apparent after discharge. It must be emphasised that only 2 patients in our experience (about 1%) had complications after an early discharge, thus demonstrating that early discharge is safe in patients who meet specific clinical criteria – mostly, whose postoperative recovery is smooth and quick.

Invariably, there will be some patients who develop complications after discharge. Is it going to create clinical or medico-legal problems? Most of complications will arise within day 3 [24], but many patients will still develop complications after day 3, that is, eventually after their discharge. We believe that an expedited and “enhanced” recovery would allow the surgeons to identify precisely – and discharge earlier - those who are less likely to develop complications so the risk of developing complications after discharge is quite low. However, also in this case, as demonstrated by our experience and in accordance with the Literature, the outcome of readmitted patients is not different from the outcome of non-readmitted ones [25], so the place where a complication becomes evident – either hospital or home – doesn’t change the final outcome.

We feel it is crucial to develop specific criteria for discharge. The old-fashioned time criterion, allowing discharge only on a specific postop day, must give way to clinical or biochemical criteria. Up until now, the decision is still with the surgeon and every single unit must develop their own discharge protocol. In our opinion, clinical criteria are still much more reliable than biochemical ones, in particular in the early postoperative period. In fact, a patient whose physical signs (temperature, blood pressure, heart rate, O2-saturation, GCS score, bowel sounds) are normal and whose postoperative pain is well controlled, can be discharged safely also when his or her inflammatory markers can still be physiologically raised due to the surgical trauma. In our opinion it is not mandatory to wait for a full bowel motion before discharging a patient; in the majority of cases, demonstration of normal bowel sounds and, possibly, passage of wind are signs of a good intestinal recovery. Finally, it is important that the patient has a direct and easy access to the surgical team at any time and that a continuous communication (and daily followup) with the patient is guaranteed by the surgical team at least for the first few days after discharge.

A complete and full involvement of the patient within the ER programme is mandatory since the beginning, also to reduce the clinical and medico-legal risk of complications. In our unit, the ER protocol is offered to any colorectal patient at their first visit and all the various components including discharge criteria are discussed and agreed with the surgeon, the anaesthetist and the specialist nurse.

One of the central components of ER protocol is a mini-invasive operation [26]. Laparoscopy fits perfectly with the philosophy of ER of reducing the physiological impact of surgery [10]. The two items, laparoscopy and ER, concur to improve the whole clinical experience of the patient. Our study confirmed that morbidity and LOS are significantly reduced in laparoscopy within an ER protocol [27], due to reduced metabolic impairment and tissue damage [28], with respect to the traditional perioperative protocols. In our series, leak rate was significantly lower in patients operated on by laparoscopy. We must admit that, from our data, it is not perfectly clear if laparoscopy itself reduces the risk of leak due to a more accurate technique or rather this reflect a ‘natural’ selection of patients as the most difficult cases – more prone to complications - ended up being operated on by open surgery.

We believe that the benefits of an ER programme are more evident in patients operated on by laparoscopy and as elective cases. For this reason, we decided to offer laparoscopic resection to all elective patients, with no other selection than the presence of an expert and skilled laparoscopic colorectal surgeon. As expected, this caused a slightly higher risk of conversion in our series with respect to the national average [19], but the rate of laparoscopic resections on the total is more than acceptable, mostly in elective cases (about 75%). Obviously, the full potential of ER protocols can be fulfilled only in elective cases, as emergency patients miss the preoperative phase of the ER protocol and can hardly be operated on by laparoscopy. However, they may still benefit from the application of some of the ER principles [6, 7], in particular as regards preoperative optimisation, fluid control, pain control and reduced invasivity. In our experience, all the emergency colectomies have been performed by open surgery and this can justify their longer postoperative stay. Our attitude to optimise as much as possible the general conditions of emergency patients before the surgical operation can be accountable for the absolutely acceptable rates of mortality, morbidity, readmission and unplanned reoperation in this subgroup, which do not differ significantly from those of the elective cases.

## Conclusions

We believe that ER protocols must become an integral part of the guidelines for the perioperative management of colorectal patients. Essential component of ERAS must be considered the laparoscopic approach, whose reduced invasiveness allows a prompt recovery, with less pain, improved mobility and possibility to identify easier the early signs of ongoing complication. An early discharge does not increase the risk of complications and does not worsen the outcome of those patients who complicate after discharge, if early discharge is included into a system that guarantees easy and prompt access to review and treatment if necessary. Specific criteria for discharge, based on clinical data, must be developed at local level and possibly endorsed by national and international guidelines. Involvement of the patients is crucial in order to get the highest level of benefit within an ERAS programme.

## Abbreviations

ER: Enhanced recovery
GCS: Glasgow coma scale
LOS: Length of hospital stay
RCT: Randomised controlled trial
SD: Standard deviation
